# Efficient Carrier Multiplication Beyond Mo- and W‑Based Transition Metal Dichalcogenides: Multilayer HfSe_2_ and ReSe_2_

**DOI:** 10.1021/acs.nanolett.6c04321

**Published:** 2026-10-29

**Authors:** Goutam Ghosh, Stevie Furxhiu, Pieter Schiettecatte, Peter Schall, Pieter Geiregat, Laurens D. A. Siebbeles

**Affiliations:** † Chemical Engineering Department, 2860 Delft University of Technology, Van der Maasweg 9, 2629 HZ Delft, The Netherlands; ‡ Physics and Chemistry of Nanostructures, Department of Chemistry, Ghent University, Ghent 9000, Belgium; § NoLIMITS Center for Non-Linear Microscopy and Spectroscopy, Ghent University, Ghent 9000, Belgium; ∥ Institute of Physics, University of Amsterdam, Amsterdam 1098 XH, The Netherlands

**Keywords:** Carrier multiplication, excitons, transition metal dichalcogenide, transient absorption spectroscopy, density functional theory

## Abstract

Transition metal dichalcogenides (TMDCs) are layered van der Waals semiconductors that can convert a single high-energy photon into multiple electron–hole pairs through carrier multiplication (CM). However, the factors governing CM efficiency within the TMDC family remain unclear, with studies limited to Mo- and W-based compositions. Here, we address this question by investigating HfSe_2_ and ReSe_2_, two less-explored TMDCs with markedly different electronic densities of states. Combining first-principles calculations with ultrafast optical spectroscopy, we establish the occurrence of efficient CM in both materials, with quantum yields 20–30% higher than those of previously studied multilayer TMDCs. Our analysis reveals that CM outcompetes phonon-mediated cooling. Furthermore, we observe similarly high CM efficiencies in HfSe_2_ and ReSe_2_ despite their vastly different number of possible CM pathways. This comparison reveals that the CM efficiency is mainly determined by the presence of at least a single CM decay pathway rather than the total number.

## Graphical abstract

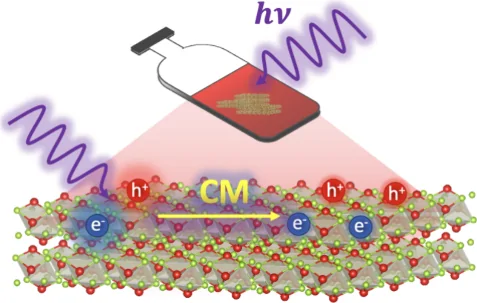

Carrier multiplication (CM), in which a single high-energy photon generates two or more electron–hole pairs, offers a direct route to overcome thermal energy losses in photoexcited semiconductors and to enhance the efficiency of solar cells and photodetectors.^1^ It provides a potential pathway to surpass the Shockley–Queisser limit in solar cells, by utilizing the excess energy of initially hot charge carriers to excite additional carriers across the band gap, rather than cooling by phonon emission.^2,3^ The efficiency of CM is governed by the competition between Coulomb-mediated carrier–carrier scattering and ultrafast carrier cooling through electron–phonon interactions. Consequently, materials with strong Coulomb interactions, high densities of electronic states, favorable band gaps, and slow carrier cooling are expected to exhibit efficient CM.^1^

Two-dimensional transition metal dichalcogenides (TMDCs) have been found to exhibit efficient CM, as reported for monolayer MoS_2_ ^4,5^ and MoSe_2_,^6^ as well as for few-layer 2H-MoSe_2_, 2H-MoTe_2_, and 2H-WSe_2_.^7–11^ Interestingly, engineered defect states in monolayer MoS_2_ and WS_2_ have enabled exceptionally low CM thresholds.^5,12^ In our previous work on solution-processed 2H-MoSe_2_ thin films, we observed efficient CM and defect-assisted processes that led to CM even below the conventional energy threshold of twice the band gap.^7^ Wang et al. recently extended such studies to twisted van der Waals heterobilayers, reporting CM with thresholds as low as twice the type-II band gap and the formation of long-lived interlayer excitons with nanosecond-scale lifetimes.^13^ These observations suggest that subtle differences in band structure, density of states, and carrier scattering pathways critically determine CM efficiency in TMDCs. Despite these advances, CM efficiency and threshold behavior remain highly material-dependent within the TMDC family and are not yet fully understood. This motivates the exploration of less-studied TMDCs with distinct electronic structures and high densities of states, such as HfSe_2_ and ReSe_2_ (see Figure 1), which are expected to provide favorable conditions for efficient carrier multiplication.

**Figure 1:**
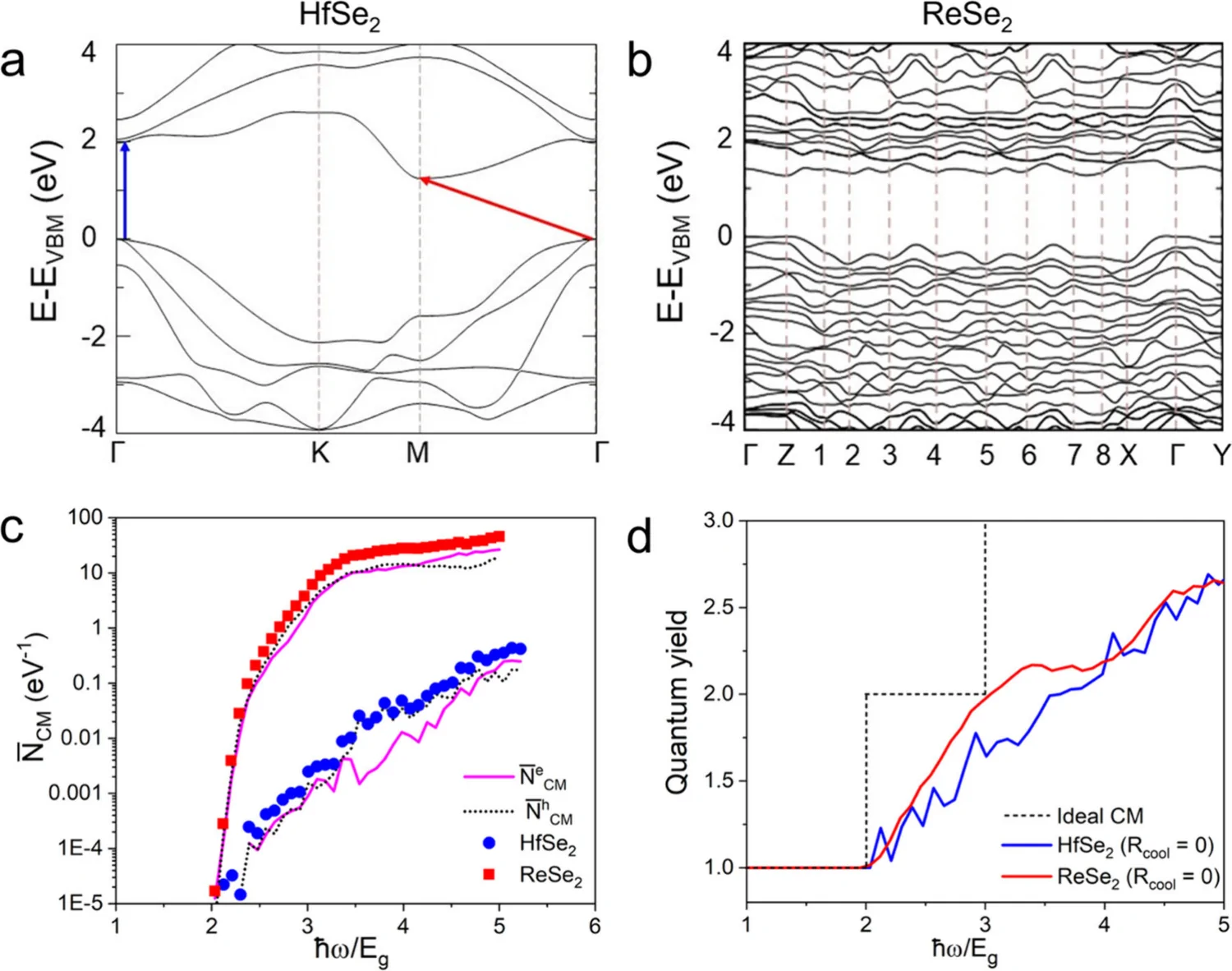
Calculated band structures and CM quantum yields. (a) Band structure of bulk 1T-HfSe_2_ with direct (blue arrow) and indirect (red arrow) band gaps indicated. (b) Band structure of 1T′-ReSe_2_. (c) Calculated total summed average density of CM pathways, *N̅*_*CM*_, along with individual contributions from electrons (*N̅*_*CM*_^*e*^) and holes (*N̅*_*CM*_^*h*^), as a function of band gap multiple (*ℏω*/*E*_*g*_) for HfSe_2_ (blue circles) and ReSe_2_ (red squares). (d) Calculated quantum yield (QY) in the absence of carrier cooling for HfSe_2_ (blue) and ReSe_2_ (red) as a function of band gap multiple. The black dashed line represents the case of ideal stair-like CM.

Here, we combine theoretical modeling and ultrafast spectroscopy to investigate carrier multiplication in solution-processed multilayered HfSe_2_ and ReSe_2_. Using our previously developed and experimentally verified theoretical model, we predict the CM efficiency from their electronic band structures and density of CM pathways. To investigate the validity of the theoretical model, we performed experiments on CM using ultrafast transient optical absorption spectroscopy. Our results demonstrate efficient CM in both materials, with quantum yields exceeding those reported for conventional TMDC materials.

Figures 1a and 1b represent the electronic band structures of bulk 1T-HfSe_2_ and 1T′-ReSe_2_, respectively, obtained from density functional theory (DFT) calculations (see Supporting Information) for bulk materials. Results for bulk are also appropriate for the few-layered samples studied experimentally in this work, since the effect of layer number on the band structure is negligible, as reported previously.^14,15^ Both materials have an indirect band gap, and we applied the scissor operator to match the experimental values of 1.13 and 1.18 eV for HfSe_2_ and ReSe_2_, respectively.^16,17^ The indirect band gap (from Γ to M) and the direct gap (at Γ) of HfSe_2_ are indicated in Figure 1a by a red and blue arrow, respectively. The density of states of HfSe_2_ is lower than for more common TMDCs (MX_2_, with M = Mo, W and X = S, Se, Te),^18–20^ while the density of states of ReSe_2_ is much higher than the Mo- and W-based TMDCs. Note that the bands of ReSe_2_ are relatively flat compared with those of HfSe_2_.

To estimate the carrier multiplication quantum yield, we calculated the number of scattering pathways that result in carrier multiplication for all initial electron and hole Bloch-states that can be produced upon photon absorption as a function of photon energy, *ℏω*. This allows us to obtain the total summed average density of CM pathways, *N̅*_*CM*_, along with individual contributions from electrons (*N̅*_*CM*_^*e*^) and holes (*N̅*_*CM*_^*h*^), as a function of band gap multiple (*ℏω*/*E*_*g*_) (see Figure 1c). Here, *N̅*_*CM*_ = *N̅*_*CM*_^*e*^ + *N̅*_*CM*_^*h*^ = $\frac{1}{N_{h\omega }}\overset{N_{h\omega }}{\underset{i=1}{\sum }}\left[N_{CM}^{e_{i}}+N_{CM}^{h_{i}}\right]$ . Details of the calculations can be found in the Supporting Information and in our previous works.^7,21^ The values of ${\mathrm{N̅}}_{CM}$ for ReSe_2_ are considerably larger and increase more rapidly than those for HfSe_2_, which can be assigned to the higher density of states of ReSe_2_, cf. Figures 1a and 1b. Interestingly, for both HfSe_2_ and ReSe_2_ the individual contributions of electrons ( ${\mathrm{N̅}}_{CM}^{e})$ and holes ( ${\mathrm{N̅}}_{CM}^{h}$ ) to the total ${\mathrm{N̅}}_{CM}$ are similar. This is different from the case for MoSe_2_, where electrons were found to dominate.^7^ When CM competes with the cooling of charge carriers via phonon emission the CM quantum yield increases with ${\mathrm{N̅}}_{CM}$ , which explains the much more efficient CM in MoTe_2_, as compared to PbS and PbSe.^21^ In contrast, if the cooling rate is negligible compared to the CM rate, then CM occurs as soon as *N̅*_*CM*_^*e*^ ≥ 1 or *N̅*_*CM*_^*h*^ ≥ 1, and a further increase of these values does not enhance the CM quantum yield. In our calculations of CM quantum yields we (1) consider pristine materials, (2) neglect cooling of charge carriers by phonon emission, (3) count CM events between initial and final electronic Bloch-states for which quasi-momentum and energy are conserved, and (4) suppose that the Coulomb coupling matrix element involving the electronic states is always nonzero. In that case, the CM quantum yield is determined by the sum of the fractions of photoexcited electrons and holes that can decay by at least one CM pathway.^7,21^ The neglect of cooling is justified by previous findings for MoTe_2_ where we found to have an effect of cooling has minor effect on CM quantum yield.^21^ In case photoexcitation does not preferentially occur to states from which CM is impossible we obtain an upper limit for the CM quantum yield of a pristine material. The CM quantum yields obtained this way are shown in Figure 1d for HfSe_2_ and ReSe_2_. Although ${\mathrm{N̅}}_{CM}$ for HfSe_2_ is orders of magnitude smaller than for ReSe_2_, the CM quantum yield of HfSe_2_ is close to that of ReSe_2_. This can be attributed to the fact that the fractions of electrons and holes that can undergo CM by at least one pathway (*N̅*_*CM*_^*e*^ ≥ 1, *N̅*_*CM*_^*h*^ ≥ 1) is similar for both materials. Nevertheless, our theoretically predicted quantum yield of ReSe_2_ is somewhat higher than that of HfSe_2_, which must be due to a higher fraction of photoexcited electrons and holes being able to decay via CM. To assess the validity of this finding, we studied the CM efficiency by ultrafast optical pump–probe spectroscopy as described below.

Powdered samples of 1T-HfSe_2_ and 1T′-ReSe_2_ were exfoliated in *N*-methyl-2-pyrrolidone (NMP) by liquid-phase exfoliation, producing predominantly multilayer, bulk-like flakes of 1T-HfSe_2_ and 1T′-ReSe_2_, as corroborated by Raman spectroscopy (see Supporting Information Figure S5). Surface oxidation of the samples is insignificant, as inferred from X-ray photoelectron spectroscopy (see Supporting Information Figure S6 and discussion). The fraction of light absorbed (F_A_) is shown in the steady-state spectra in Figures 2a and 2b as a function of photon energy. The absorption of HfSe_2_ has an onset near 1.8 eV and then rises smoothly. Interestingly, we did not observe any sharp exciton peak as usually observed in other transition metal dichalcogenides (TMDCs),^7^ probably because of inhomogeneity in the sample. By contrast, the absorption spectrum of ReSe_2_ shows a shoulder near 1.3 eV, which is likely due to excitonic transitions.

**Figure 2:**
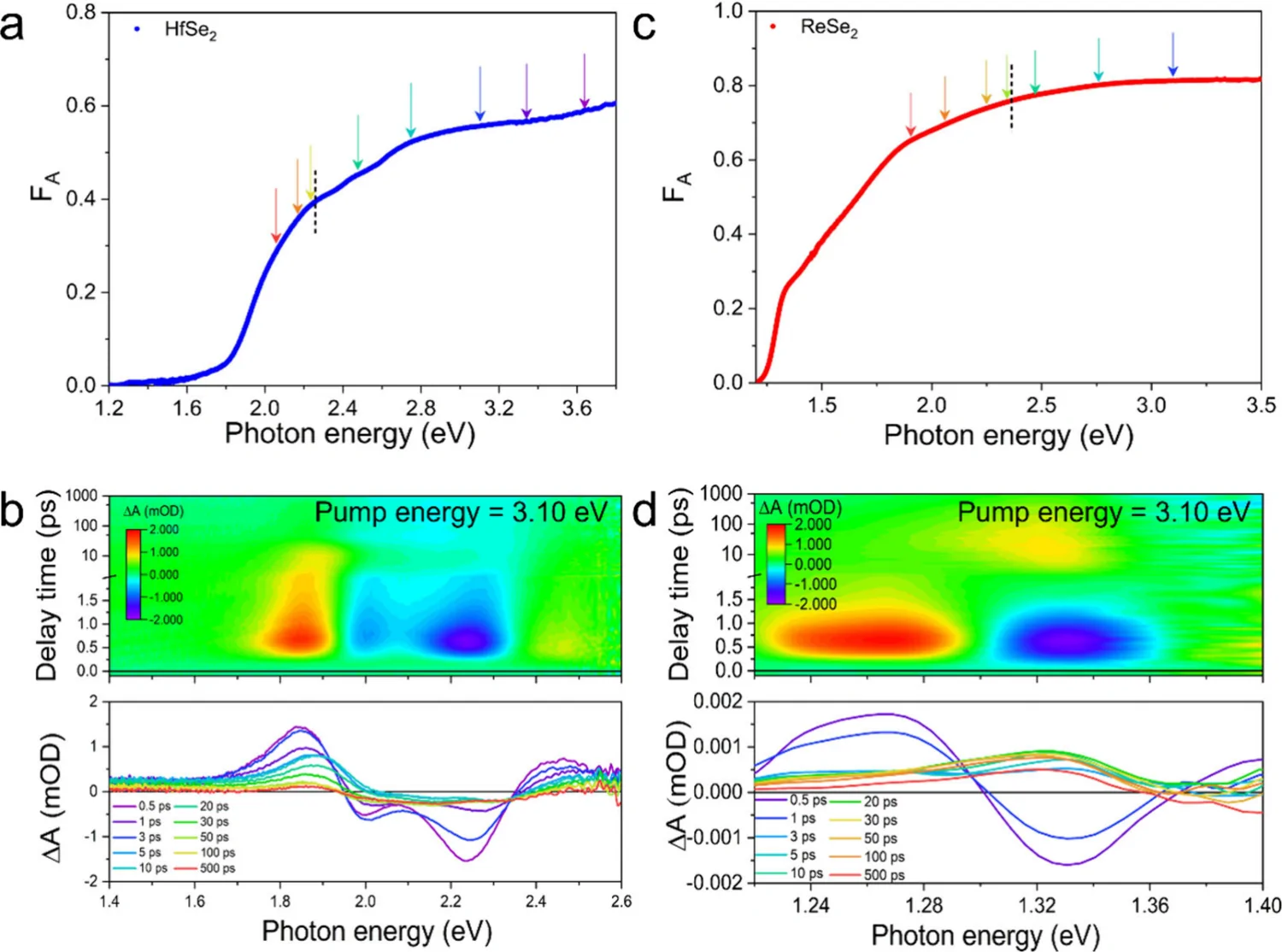
Steady-state and transient absorption (TA) spectroscopy. (a, c) Fraction of light absorbed by (a) HfSe_2_ and (c) ReSe_2_. The vertical dotted line indicates twice the band gap energy. Colored arrows indicate the photon energies at which the quantum yield of electron–hole pairs was evaluated. (b, d) TA colormaps (upper panels) and corresponding TA spectra at the selected delay times (lower panels) of (b) HfSe_2_ and (d) ReSe_2_ at 3.10 eV pump photon energy and photon fluence values of 37 × 10^12^ and 42 × 10^12^ photons/cm^2^, respectively.

We studied the photogeneration and decay dynamics of excitons and charge carriers using transient optical absorption (TA) spectroscopy (see Supporting Information), as detailed previously.^7,22^ An ultrashort pump pulse with tunable photon energy generates electron–hole pairs, which relax toward the band edges and eventually recombine. A broadband white-light pulse is used as the probe to monitor the pump-induced change of optical density, Δ*A*(*E*, τ), as a function of probe photon energy (*E*) and pump–probe delay time (τ). The TA spectrum is obtained as Δ*A*(*E*, τ) = $\log_{10}\left(\frac{I_{off}^{probe}\left(E\right)}{I_{on}^{probe}\left(E,\tau \right)}\right)$ , where *I*_*on*_^*probe*^ and *I*_*off*_^*probe*^ are the amounts of transmitted probe-light photons with and without photoexcitation, respectively.^23,24^ The pump and probe pulses each have a duration of ∼180 fs, leading to a time resolution of ∼0.25 ps. Therefore, we only considered our spectral analysis to the TA spectra at delays ≥0.5 ps.

Figures 2c and 2d display the TA colormap (upper panel) along with TA spectral slices (lower panel) at different pump–probe delay times, following excitation with a pump photon energy of 3.10 eV for HfSe_2_ and ReSe_2_. For HfSe_2_ the TA spectra at early times show a pump-induced absorption just above 1.8 eV and two distinct bleach signals near 2.0 and 2.23 eV followed by transient absorption above 2.4 eV. Such derivative-like absorption and bleach features are typical for a shift of the probe spectrum after generation of charge carriers or excitons by the pump pulse.^7,22,24^ The probe pulse then generates trions or biexcitons with absorption features that are red-shifted from the original exciton peaks.^25^ A negative TA signal may also have contributions of probe-induced emission from excitons. Up to a delay time of 3 ps the TA spectra exhibit pronounced spectral evolution, while their shape becomes merely constant after 10 ps. These early time spectral modifications arise from the relaxation of the initially generated hot electron–hole pairs to the band edges or exciton states.^25,26^ The shapes of the TA spectra of ReSe_2_ in Figure 2d also exhibit significant changes at early delay times up to about 10 ps due to relaxation of initially hot electrons and holes. At early times up to 1 ps both induced absorption and bleach are observed, while after 20 ps only a broad absorption centered around 1.33 eV is present. Our results on charge carrier relaxation dynamics on the order of several picoseconds agree with those for other TMDCs.^8,27,28^ After 20 ps the spectral shape of the TA spectra remains essentially unchanged, while the overall TA amplitude decreases over time due to electron–hole recombination. The TA amplitude at longer times thus reflects the population of charge carriers and excitons. The decay kinetics are independent of pump fluence, or equivalently, initial density of charge carriers and excitons (see Figures S1 and S2). This means that charge carriers and excitons decay by first-order kinetics due to trapping or geminate recombination, rather than e.g. second-order radiative charge recombination, nonradiative exciton–exciton annihilation, or third-order Auger recombination of charge carriers. The CM quantum yield is obtained from the TA amplitude at different pump photon energies and fluences as discussed below.

The quantum yield, Φ, is defined as the initial number of photogenerated pairs of free electrons and holes plus the number of excitons per absorbed pump photon. The TA amplitude is given by Δ*A*(*E*, τ) = Φ*I*_0_ *F*_*A*_ *s*(τ)­Δσ­(*E*)/ln­(10), where *I*_0_ *F*_*A*_ is the absorbed pump fluence, *s*(τ) is the survival fraction of electron–hole pairs at time τ after the pump pulse, and Δσ­(*E*) is the change of the absorption cross section at probe photon energy *E*.^1,7,29^ The variation of the ratio ΔA/*I*_0_ *F*_*A*_ with pump photon energy thus reflects the change of Φ due to CM.

Figure 3 shows the normalized TA spectrum, Δ*A*/*I*_0_ *F*_*A*_, for HfSe_2_ and ReSe_2_ at different pump photon energies. To enhance the signal-to-noise ratio we averaged Δ*A* over a time delay interval of τ = 20–50 ps, similar to previous studies.^7,29^ As seen can be seen in Figure 3a, the shape of the normalized TA spectrum is similar for all photon energies, while its amplitude increases. We attribute the increase in the amplitude to the occurrence of CM, which enhances the quantum yield, Φ. The finding that the shape of the TA spectra does not significantly change with quantum yield, agrees with the similar shapes of TA spectra for different pump fluences (see Figure S3). The amplitudes of the normalized TA spectra at the two lowest pump photon energies in Figure 1a are the same, which implies the same quantum yields. This can be understood, since both photon energies are below (or just at) twice the band gap (2*E*_*g*_ = 2.26 eV) of HfSe_2_ and CM does not occur. The amplitude increases at higher pump photon energies that exceed twice the band gap, which is due to CM. The amplitudes of the normalized TA spectra for ReSe_2_ in Figure 3b are similar for the two lowest pump photon energies, which are below twice the band gap energy (2*E*_*g*_ = 2.36 eV). At higher pump photon energy, the amplitude increases, indicating the occurrence of CM.

**Figure 3:**
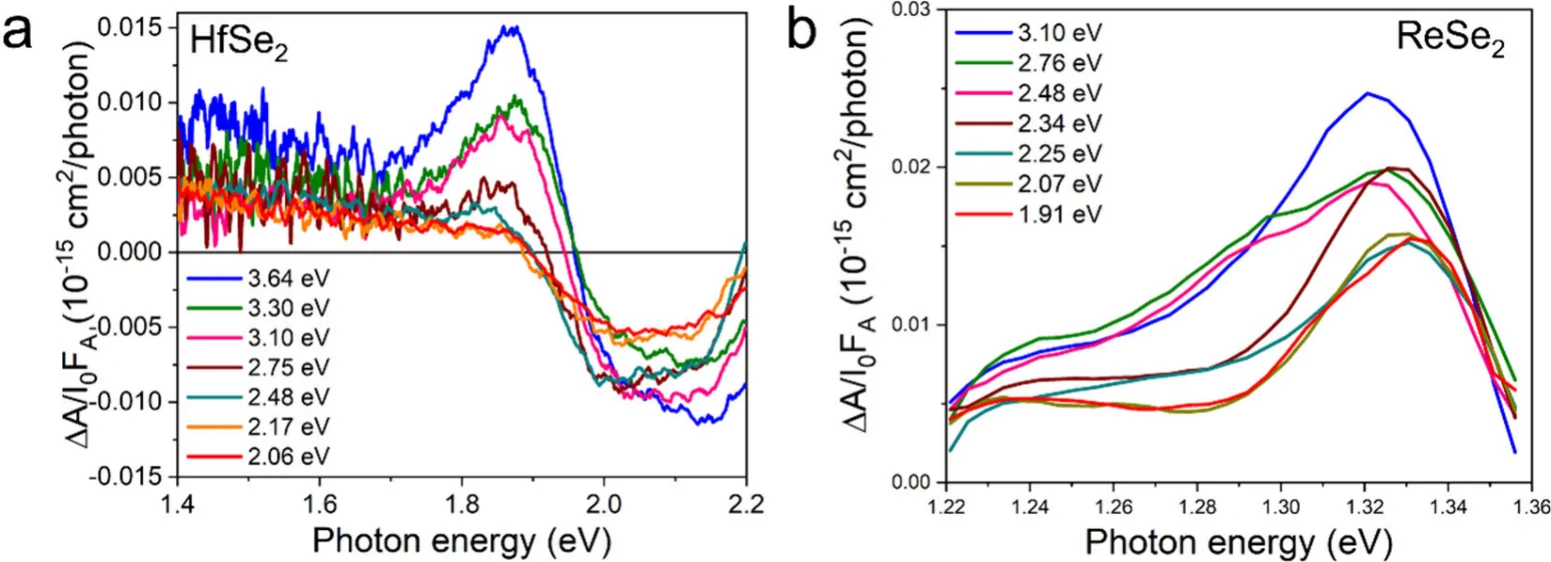
Signatures of CM. (a, b) Transient change in optical absorption normalized to the absorbed pump fluences, (9–10) × 10^12^ photons/cm^2^, and averaged over delay times in the interval 20–50 ps for pump photon energies as indicated for (a) HfSe_2_ and (b) ReSe_2_. The increase in the magnitude of the normalized TA spectra indicates the occurrence of CM.

Note that the shape of the TA spectra of ReSe_2_ in Figure 3b varies with pump photon energy, or equivalently with the density of charge carriers and/or excitons. This is further demonstrated in Figure 4, which shows that the shape of the normalized TA spectra of ReSe_2_ varies with the fluence of pump photons, both with an energy of 1.91 eV (below CM threshold) and 3.1 eV (above CM threshold). Interestingly, an isosbestic point appears at a probe photon energy of 1.305 eV. At probe energies below this value, the normalized TA magnitude increases with pump fluence, while it decreases at higher probe energies. The presence of such isosbestic point is typical for interconversion between two distinct photogenerated species, which have the same optical absorption cross section at the isosbestic photon energy. We attribute the TA signal on the lower-energy side of the isosbestic point to contributions from excitons, since their quantum yield increases with the density of photoexcitation due to enhanced recombination of free electrons and holes. This agrees with the decrease in the TA signal due to charge carriers with photoexcitation density, which appears at the high-energy side of the isosbestic point. At the probe photon energy of the isosbestic point the absorption cross sections of excitons and charge carriers are equal. Hence, at this probe energy the magnitude of the TA signal is proportional to the total population of excitons and free charge carriers.

**Figure 4:**
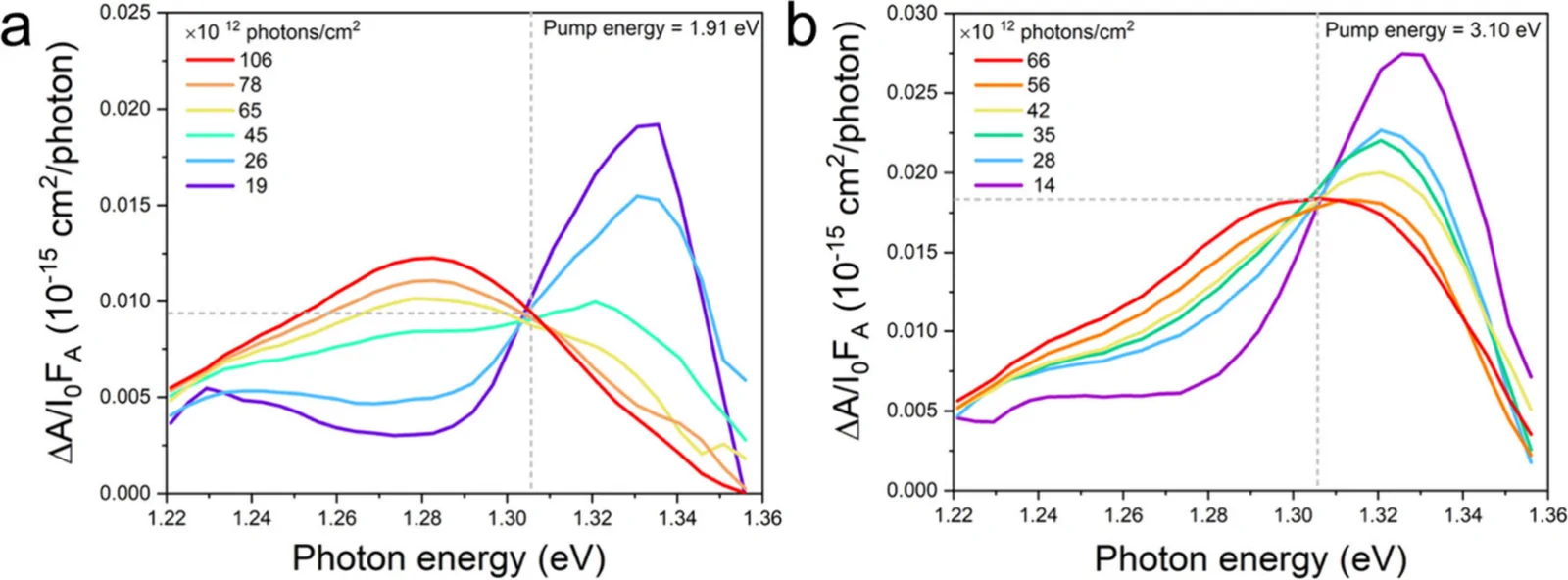
Transient change in optical absorption of ReSe_2_ normalized to the absorbed pump fluence and averaged over a delay time range of 20–50 ps for a pump photon energy of (a) 1.91 eV and (b) 3.10 eV. The magnitude of the TA signal increases at low probe photon energies and decreases at higher energies. The appearance of an isosbestic point is attributed to the conversion of free charge carriers into excitons as the pump fluence increases. The occurrence of CM at a pump energy of 3.10 eV causes the TA signal at the isosbestic point to be about twice as high as that for a pump energy of 1.91 eV.

Indeed, Figure 4 shows that the magnitude of the normalized TA signal at the isosbestic point is independent of pump fluence. Interestingly, the magnitude of the normalized TA signal for 3.10 eV pump photon energy (Figure 4b) is a factor of 2 higher than for 1.91 eV, which is below the 2*E*_*g*_ CM threshold (Figure 4a). This directly reflects the occurrence of CM at 3.10 eV.

We use the magnitude of the TA signal at the isosbestic point to determine the CM quantum yield in ReSe_2_, whereas for HfSe_2_, we use the absolute value of the TA amplitude integrated over probe photon energy, as in previous studies.^1,7,29^ To enhance the signal-to-noise ratio we average the TA spectra over 20–50 ps delay times. To determine the CM quantum yield for HfSe_2_ we additionally calculate the integrated absolute TA signal, $\overset{®}{\Delta A}$ , by summing the absolute values of Δ*A*(*E*) over the entire probe photon region, as in our previous studies.^1,7,29^ For ReSe_2_ the latter integration is not appropriate, since the shape of the TA spectrum depends on the density of charge carriers and excitons, as discussed above. Therefore, we determine the CM quantum yield from the amplitude at the probe photon energy of the isosbestic point, Δ*A*_*iso*_, in the TA spectrum, which reflects the total density of excitons and free charge carriers, since they have the same absorption cross section at this energy.

Figure 5a shows $\overset{®}{\Delta A}$ for HfSe_2_ as a function of absorbed photon fluence for different pump photon energies. The linear increase of $\overset{®}{\Delta A}$ with fluence indicates that higher-order recombination processes of excitons or charge carriers is negligible. This interpretation is further supported by the absence of any fluence dependence in the decay kinetics of the TA signal probed at 1.86 eV after 3.10 eV excitation (see Figure S1). For each photoexcitation energy in Figure 5a, the fluence dependence of $\overset{®}{\Delta A}$ was fitted linearly to obtain the slope, which is directly proportional to the quantum yield, Φ, as discussed above. Therefore, an increase in the slope with pump photon energy reflects an increase in the quantum yield. The slopes for the lowest two pump photon energies (2.06 and 2.17 eV) below twice the band gap, are the same within the experimental uncertainty and reflect the signal for quantum yield equal to one. Figure 5c shows the CM quantum yield obtained from the slopes in Figure 5a as a function of band gap multiple, *ℏω*/*E*_*g*_, (bottom axis) and pump photon energy, *ℏω*, (top axis). The measured CM quantum yields for HfSe_2_ start to increase at a photon energy equal to twice the band gap and exceed the value of two at three times the band gap energy.

**Figure 5:**
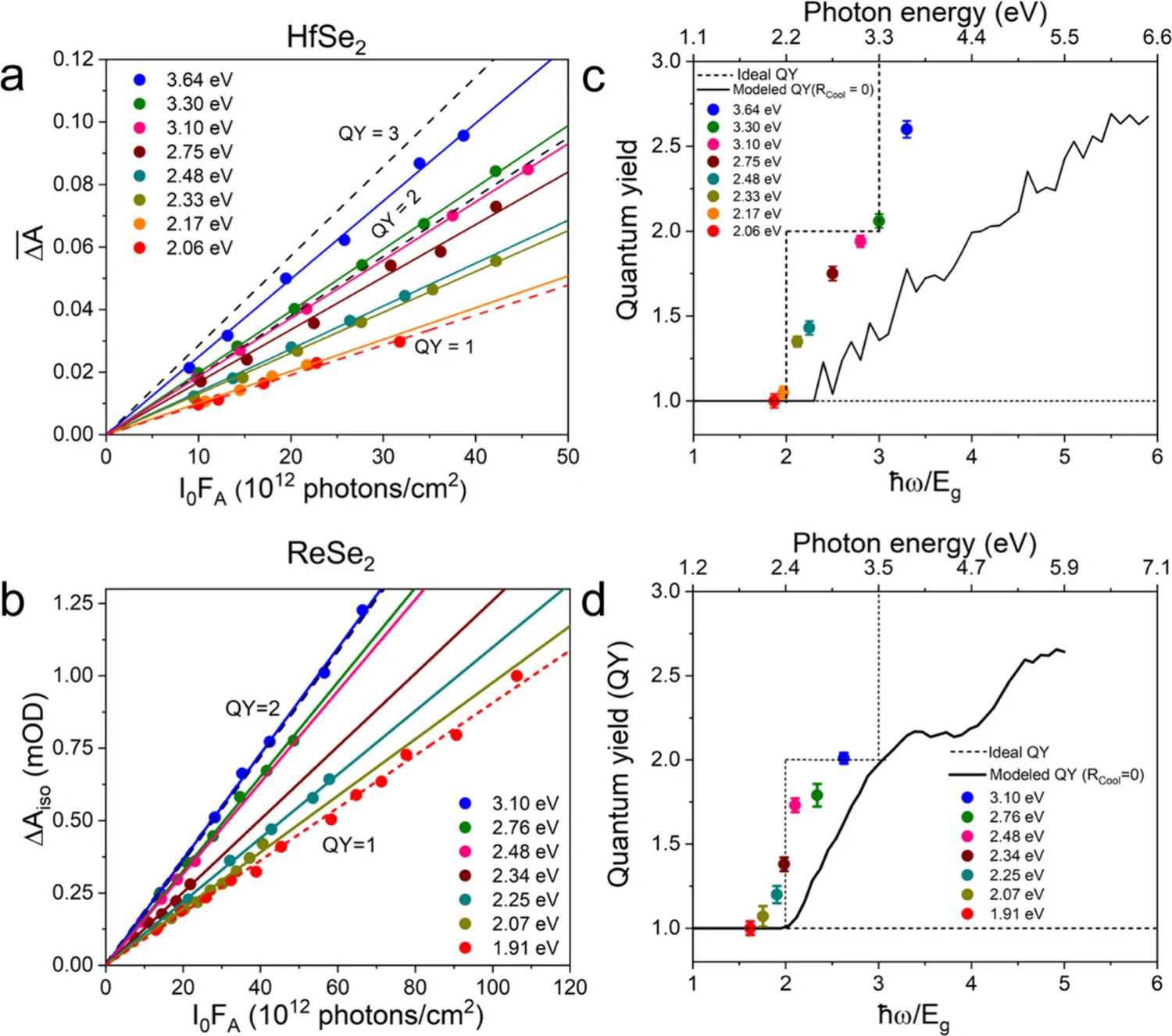
Determination of CM quantum yield. (a) Integrated absolute TA signal, $\overset{®}{\Delta A}$ , for HfSe_2_ as a function of absorbed photon fluence for different pump photon energies. (b) TA signal at the isosbestic point, Δ*A*_*iso*_(*E* = 1.305 eV), for ReSe_2_, plotted against absorbed photon fluence for various pump photon energies. Solid lines are linear fits to the experimental data, with their slopes representing the quantum yields. (c, d) Quantum yield as a function of band gap multiple (bottom axis) and pump photon energy (top axis) for (c) HfSe_2_ and (d) ReSe_2_. The black dashed lines represent the case of ideal stair-like CM, and the black solid curves are the calculated quantum yields.

For ReSe_2_, the amplitude at the isosbestic point, Δ*A*_*iso*_(*E* = 1.305 eV), is plotted as a function of absorbed pump photon fluence for different pump photon energies (see Figure 5b). The linear increase of Δ*A*_*iso*_ with fluence rules out higher-order recombination processes. This conclusion is further supported by the absence of any fluence dependence on the TA decay kinetics probed at the isosbestic point for excitation energies of 1.91 and 3.10 eV (see Figure S2). Figure 5d shows the CM quantum yield obtained from the slopes in Figure 5b. The quantum yield begins to exceed 1 at a pump photon energy of 2.07 eV (*ℏω*/*E*_*g*_ > 1.75), then continues to increase, approaching a value of nearly 2 at 3.10 eV. Notably, in ReSe_2_ the quantum yield exceeds 1 at a photon energy below the standard CM threshold of twice the band gap. Such low-threshold CM has been reported previously for other TMDCs and was attributed to the involvement of subgap states due to defects such as chalcogen vacancies, or changes in band structure due to electron–phonon coupling.^5,7,12,30,31^ According to previous studies, Se vacancies are present in ReSe_2_ and give rise to subgap states. These are likely present in our sample as well, and will facilitate CM below the threshold of twice the band gap.^32,33^

A comparison of Figures 5c and 5d shows that the experimental CM quantum yields for ReSe_2_ are somewhat higher than for HfSe_2_, in qualitative agreement with the calculated results. The difference in CM quantum yields for the two materials is much smaller than that of their average density of CM pathways, ${\mathrm{N̅}}_{CM}$ (see Figure 1c). This leads to the conclusion that hot charge carrier cooling via phonon emission is negligible, and that the fraction of photoexcited electrons and holes with at least one CM decay pathway is similar in both materials. Furthermore, from the fact that our experimental CM quantum yields are about 0.6 ± 0.3 higher than the calculated values, we conclude that subgap defects and band gap reduction due to electron–phonon coupling facilitate CM. This is quantitatively supported by the experimental enhancement of the quantum yield from 1.0 to 1.4 for photon energies below twice the band gap by deliberate introduction of 1.5% S-vacancies in monolayer MoS_2_.^5^ In addition, according to calculations the quantum yield in TMDCs can be further enhanced by an amount of about 0.05 due electron–phonon coupling.^30^

The CM quantum yields as a function of band gap normalized photon energy for our multilayer bulk-like HfSe_2_ and ReSe_2_ samples are 20–30% higher than those reported for multilayered or bulk MoTe_2_,^8–10^ MoSe_2_,^6,7^ and MoS_2_/WSe_2_ heterobilayers.^13^ It is noteworthy that our quantum yields for ReSe_2_ are comparable to those for monolayer MoS_2_^4^ and MoSe_2_, which however have much lower absorption due to their lower thickness.^6^

In summary, we studied the efficiency of CM in multilayered HfSe_2_ and ReSe_2_ using a combination of theory and TA measurements. By comparing the calculated and experimental results, we infer that the rate of CM exceeds the rate of carrier cooling by phonon emission. The CM efficiencies in HfSe_2_ and ReSe_2_ are similar, despite the much larger density of CM pathways in ReSe_2_. This implies that the CM efficiency is mainly determined by the presence of at least a single CM decay pathway rather than the density of pathways. Even though our theoretical model is supposed to provide an upper limit of the CM quantum yields of a pristine material, the calculated values are lower than the experimental ones. We attribute this to subgap defects or to band gap reduction due to electron–phonon coupling. Interestingly, the CM quantum yields of our HfSe_2_ and ReSe_2_ samples are higher than those reported previously for other multilayer TMDCs. This offers promising prospects for their application in solar cells or photodetectors.

## Supplementary Material



## Data Availability

The authors declare that the data supporting the findings of this study are available within the paper and its Supporting Information. Data are also available from the corresponding authors upon reasonable request.

## Notes

Details of methods for electronic band structure calculations, carrier multiplication modeling, material synthesis, and sample characterization, as well as additional TA decay kinetics measured at different pump fluences and photon energies for HfSe_2_ and ReSe_2_, normalized TA spectra for HfSe_2_, TEM images, Raman spectra, and XPS spectra (PDF)

## References

[1] Maiti S., van der Laan M., Poonia D., Schall P., Kinge S., Siebbeles L. D. A. (2020). Emergence of New Materials for Exploiting Highly Efficient
Carrier Multiplication in Photovoltaics. Chem.
Phys. Rev..

[2] Shockley W., Queisser H. J. (1961). Detailed Balance
Limit of Efficiency of p-n Junction
Solar Cells. J. Appl. Phys..

[3] Hanna M. C., Nozik A. J. (2006). Solar Conversion
Efficiency of Photovoltaic and Photoelectrolysis
Cells with Carrier Multiplication Absorbers. J. Appl. Phys..

[4] Soni A., Kushavah D., Lu L.-S., Chang W.-H., Pal S. K. (2023). Efficient
Multiple Exciton Generation in Monolayer MoS_2_. J. Phys. Chem. Lett..

[5] Karmakar R., Taank P., Ghoshal D., Yadav P., Mandal D., Shrivastava M., Agarwal A., Beard M. C., Miller E. M., Adarsh K. V. (2025). Multiple
Carrier Generation at an Exceptionally Low
Energy Threshold. Phys. Rev. Lett..

[6] Kim J., Min H.-G., Park S., Park J. C., Bang J., Kim Y., Kim J.-H. (2025). Hot carrier
diffusion-assisted ideal carrier multiplication
in monolayer MoSe_2_. Mater. Horiz..

[7] Ghosh G., Carey T., Furxhiu S., Weerdenburg S., Singh N., van der Laan M., Branchett S. E., Jaspers S., Suijkerbuijk J. W., Lipilin F. (2025). Carrier
Multiplication and Photoexcited Many-Body States in Solution-Processed
2H-MoSe_2_. ACS Nano.

[8] Kim J.-H., Bergren M. R., Park J. C., Adhikari S., Lorke M., Frauenheim T., Choe D.-H., Kim B., Choi H., Gregorkiewicz T. (2019). Carrier Multiplication in Van Der Waals Layered
Transition Metal Dichalcogenides. Nat. Commun..

[9] Zheng W., Bonn M., Wang H. I. (2020). Photoconductivity
Multiplication
in Semiconducting Few-Layer MoTe_2_. Nano Lett..

[10] Robey S. W., Krylyuk S., Davydov A. V., Heilweil E. J. (2024). Optical
Pump–Terahertz
Probe Measurements of Photocarrier Multiplication in 2D Bulk Single-Crystal
2H-MoTe_2_. J. Phys. Chem. C.

[11] Kim J. S., Tran M. D., Kim S. T., Yoo D., Oh S.-H., Kim J.-H., Lee Y. H. (2021). Escalated Photocurrent with Excitation
Energy in Dual-Gated MoTe_2_. Nano
Lett..

[12] Li J., Zhang Y., Sun J.-D., Li Y., Yan C., Wang Q., Gao Y., Xin W., Fu J., Liu W. (2025). Defect-Engineered Reduction of the Carrier Multiplication
Threshold in Monolayer WS_2_ to 1.6Eg. ACS Nano.

[13] Wang P., Wang G., Wang C., Wei Q., Chen Y., Liu Q., Zhou L., Ren H., Zhang X., Peng J. (2026). Low-threshold interlayer exciton multiplication in twisted transition
metal dichalcogenides heterobilayers. Light:
Sci. Appl..

[14] Choi B. K., Ulstrup S., Gunasekera S. M., Kim J., Lim S. Y., Moreschini L., Oh J. S., Chun S.-H., Jozwiak C., Bostwick A. (2020). Visualizing Orbital Content of Electronic Bands
in Anisotropic 2D Semiconducting ReSe_2_. ACS Nano.

[15] Hua Z., Shu H., Xie D., Liu Z., Liang J., Zhou J., Chen X., Cao D. (2025). Unraveling
the Interlayer Coupling
Effect on Layer-Dependent Electronic and Optoelectronic Properties
in Two-Dimensional Semiconductors. J. Mater.
Chem. C.

[16] Jiang H. (2011). Structural
and Electronic Properties of ZrX_2_ and HfX_2_ (X
= S and Se) from First Principles Calculations. J. Chem. Phys..

[17] Ho C. H., Huang C. E. (2004). Optical Property of the Near Band-Edge Transitions
in Rhenium Disulfide and Diselenide. J. Alloys
Compd..

[18] Dybała F., Polak M. P., Kopaczek J., Scharoch P., Wu K., Tongay S., Kudrawiec R. (2016). Pressure coefficients for direct
optical transitions in MoS2, MoSe2, WS2, and WSe2 crystals and semiconductor
to metal transitions. Sci. Rep..

[19] Jones L. A. H., Xing Z., Swallow J. E. N., Shiel H., Featherstone T. J., Smiles M. J., Fleck N., Thakur P. K., Lee T.-L., Hardwick L. J. (2022). Band
Alignments, Electronic Structure, and
Core-Level Spectra of Bulk Molybdenum Dichalcogenides (MoS2, MoSe2,
and MoTe2). J. Phys. Chem. C.

[20] Jiang H. (2012). Electronic
Band Structures of Molybdenum and Tungsten Dichalcogenides by the
GW Approach. J. Phys. Chem. C.

[21] Weerdenburg S., Singh N., van der Laan M., Kinge S., Schall P., Siebbeles L. D. A. (2024). New
Theoretical Model to Describe Carrier Multiplication
in Semiconductors: Explanation of Disparate Efficiency in MoTe_2_ versus PbS and PbSe. J. Phys. Chem.
C.

[22] Schiettecatte P., Poonia D., Tanghe I., Maiti S., Failla M., Kinge S., Hens Z., Siebbeles L. D. A., Geiregat P. (2021). Unraveling the Photophysics of Liquid-Phase Exfoliated
Two-Dimensional ReS_2_ Nanoflakes. J. Phys. Chem. C.

[23] Pasanen H. P., Khan R., Odutola J. A., Tkachenko N. V. (2024). Transient
Absorption Spectroscopy of Films: Impact of Refractive Index. J. Phys. Chem. C.

[24] Schiettecatte P., Hens Z., Geiregat P. (2023). A Roadmap
to Decipher Ultrafast Photophysics
in Two-Dimensional Nanomaterials. J. Chem. Phys..

[25] Failla M., García Flórez F., Salzmann B. B. V., Vanmaekelbergh D., Stoof H. T. C., Siebbeles L. D. A. (2023). Effects
of Pump Photon Energy on
Generation and Ultrafast Relaxation of Excitons and Charge Carriers
in CdSe Nanoplatelets. J. Phys. Chem. C.

[26] Schmitt-Rink S., Chemla D. S., Miller D. A. B. (1985). Theory of Transient Excitonic Optical
Nonlinearities in Semiconductor Quantum-Well Structures. Phys. Rev. B.

[27] Steinleitner P., Merkl P., Nagler P., Mornhinweg J., Schüller C., Korn T., Chernikov A., Huber R. (2017). Direct Observation of Ultrafast Exciton Formation in a Monolayer
of WSe_2_. Nano Lett..

[28] Wang T., Hopper T. R., Mondal N., Liu S., Yao C., Zheng X., Torrisi F., Bakulin A. A. (2023). Hot Carrier Cooling
and Trapping in Atomically Thin WS_2_ Probed by Three-Pulse
Femtosecond Spectroscopy. ACS Nano.

[29] Maiti S., Ferro S., Poonia D., Ehrler B., Kinge S., Siebbeles L. D. A. (2020). Efficient Carrier Multiplication in Low Band Gap Mixed
Sn/Pb Halide Perovskites. J. Phys. Chem. Lett..

[30] Liu Y., Frauenheim T., Yam C. (2022). Carrier Multiplication in Transition
Metal Dichalcogenides Beyond Threshold Limit. Adv. Sci..

[31] Zhou W., Zou X., Najmaei S., Liu Z., Shi Y., Kong J., Lou J., Ajayan P. M., Yakobson B. I., Idrobo J.-C. (2013). Intrinsic Structural
Defects in Monolayer Molybdenum Disulfide. Nano
Lett..

[32] Zhu Y., Tao L., Chen X., Ma Y., Ning S., Zhou J., Zhao X., Bosman M., Liu Z., Du S. (2021). Anisotropic Point Defects in Rhenium Diselenide Monolayers. iScience.

[33] Lam N. H., Ko J.-H., Choi B. K., Ly T. T., Lee G., Jang K., Chang Y. J., Soon A., Kim J. (2023). Direct Characterization
of Intrinsic Defects in Monolayer ReSe_2_ on graphene. Nanoscale Adv..

