# Supplementary material for: Efficient Carrier Multiplication Beyond Mo- and W‑Based Transition Metal Dichalcogenides: Multilayer HfSe2 and ReSe2
Source: Nano Lett. 2026 Oct 29;26(44):15424–31. doi: 10.1021/acs.nanolett.6c04321 (PMC13761693; doi:10.1021/acs.nanolett.6c04321)
Supplement: Supplementary file 1 [file nl6c04321_si_001.pdf]

# Supporting Information

## Efficient Carrier Multiplication Beyond Mo- and W-Based Transition Metal Dichalcogenides: Multilayer $HfSe_2$ and $ReSe_2$

Goutam Ghosh[1, 2, 3*], Stevie Furxhiu[1], Pieter Schiettecatte[2, 3], Peter Schall[4], Pieter Geiregat[2, 3], Laurens D.A. Siebbeles[1*]

[1]*Chemical Engineering Department, Delft University of Technology, Van der Maasweg 9, 2629 HZ Delft, The Netherlands*

[2]*Physics and Chemistry of Nanostructures, Department of Chemistry, Ghent University, Ghent 9000, Belgium; Center for Nano and Biophotonics, Ghent University, Ghent 9000, Belgium*

[3]*NoLIMITS Center for Non-Linear Microscopy and Spectroscopy, Ghent University, Gent 9000, Belgium*

[4]*Institute of Physics, University of Amsterdam, Amsterdam 1098 XH, The Netherlands*

## METHODS

**Electronic Band Structure Calculations.** Electronic band structures were calculated using Density Functional Theory (DFT), as implemented in the ABINIT package.[1] The calculations were carried out using norm-conserving fully relativistic pseudopotentials based on the generalized gradient approximation for the exchange–correlation functional according to Perdew-Becke-Ernzerhof. The lattice constants for hexagonal $HfSe_2$ were taken as $a$ = 3.74 Å and $c$ = 6.16 Å,[2-4] while for triclinic $ReSe_2$ the lattice constants were taken to be $a$ = 6.72 Å, $b$ = 6.60 Å and $c$ = 6.71 Å.[5] For both materials a 14×14×7 $k$-point grid and an energy cut-off of 40 Hartree (~1088 eV) were used. The calculated band gaps are 0.36 eV (indirect) and 1.22 eV (direct) for $HfSe_2$, and 0.96 eV (indirect) and 0.97 eV (direct) for $ReSe_2$. The bands were scissor corrected to match the experimental indirect band gaps of 1.13 eV for $HfSe_2$ and 1.18 eV for $ReSe_2$.[6, 7]

**Modeling Carrier Multiplication (CM).** The quantum yield was calculated following the approach outlined in our earlier work,[8] with the electronic band structures from the DFT calculations described above. Briefly, we consider the CM pathways for all combinations of initial and final states of an electron or hole in the conduction and valence bands. In case a pathway involving an initial and final state conserving both energy and quasi-momentum ($k$), it contributes to the number of CM events, $N_{CM}$. By correlating the initial electron and hole states with the corresponding photon energy, we obtain the average density of CM pathways according to $\overline{N}_{CM} = \overline{N}_{CM}^{e} + \overline{N}_{CM}^{h} = \frac{1}{N_{\hbar\omega}}\sum_{i=1}^{N_{\hbar\omega}}[N_{CM}^{e_i} + N_{CM}^{h_i}]$, where $N_{CM}^{e_i}$ ($N_{CM}^{h_i}$) is the density of CM pathways of an electron (hole) for optical transition $i$, and $N_{\hbar\omega}$ is the number of allowed optical transitions for a photon with energy $\hbar\omega$. From this, we calculate the quantum yield according to $\Phi(\hbar\omega) = 1 + \frac{1}{N_{\hbar\omega}}\sum_{i=1}^{N_{\hbar\omega}} P_{CM}^{e_i} + \frac{1}{N_{\hbar\omega}}\sum_{i=1}^{N_{\hbar\omega}} P_{CM}^{h_i}$, where $P_{CM}^{e_i}$ and $P_{CM}^{h_i}$ denote the CM probability for the excited electron and hole in transition $i$. In the previous framework,[8] $P_{CM}$ depends on $N_{CM}$, a rate constant $F_{CM}$, and the carrier cooling rate $R_{cool}$, which competes with CM. Here, we neglect carrier cooling ($R_{cool} = 0$), so the calculated quantum yield represents an upper bound. Under this assumption, the CM probability for transition $i$ is defined as

$$P_{CM}^{i} = \begin{cases} 1 \text{ if } N_{CM}^{i} > 0 \\ 0 \text{ otherwise} \end{cases} .$$

**Liquid-Phase Exfoliation of $HfSe_2$ and $ReSe_2$.** $HfSe_2$ and $ReSe_2$ were exfoliated following the protocol reported by Schiettecatte *et al.*[9] and starting from commercial $HfSe_2$ and $ReSe_2$ bulk powders, respectively, obtained from supplier Ossila. Commercial $HfSe_2$ (≥99.995% purity, product code M2153C1) and $ReSe_2$ (≥ 99.995% purity, product code M2140C1) powders were stored in a nitrogen-filled glove box. For each material, 50 mg of powder was dispersed in 25 mL of N-methyl-2-pyrrolidone (NMP, ≥ 99.0%, Merck), capped under nitrogen, and sonicated for 400 min in a bath sonicator (USC-THD, VWR). During sonication, the temperature was maintained below 45 °C to prevent autoxidative transformations of NMP. The resulting dispersions were centrifuged (Eppendorf 5804) at 400g (1.5 krpm) for 60 min, after which approximately 75% of the upper supernatant was carefully decanted using a Pasteur pipette. The collected supernatant, containing exfoliated flakes, was subsequently centrifuged at 16,000g for 10 min. Finally, the sediment was redispersed in fresh N-methyl-2-pyrrolidone (NMP) for further studies. This procedure yields multilayered flakes with typical thickness of 4 ± 2 monolayers.[9]

**Transmission Electron Microscopy (TEM).** For TEM characterization, the $ReSe_2$/$HfSe_2$ dispersion in NMP was diluted with 2-propanol and drop-cast onto a holey carbon-coated Cu grid (200 mesh, 50 μm). The samples were dried overnight under vacuum to remove residual solvent and subsequently imaged using a Cs-corrected JEOL JEM-2200FS TEM operating at 200 kV. TEM characterization of both samples shows a heterogeneous distribution of flake sizes and thicknesses, with most flakes exhibiting multilayered or bulk-like structures (see Figure S4).

**Raman Spectroscopy.** Samples for Raman analysis were prepared by casting a dispersion in NMP on a glass slide, and removing the solvent under vacuum in the antechamber of a glove box. Raman measurements were performed in air. Raman measurements were conducted on a Bruker Optics Senterra dispersive Raman spectrometer attached to an Olympus BX51 microscope and coupled to a thermoelectrically cooled CCD detector, operating at -65 °C. The spectrometer has an XYZ motorised (automatic) stage for positioning and focusing. The samples were studied as dried films on glass slides. Spectra were obtained in the spectral range of 50 to 1540 $cm^{-1}$ and with a spectral resolution of 3–5 $cm^{-1}$ using a green Nd:YAG (532 nm) laser. The experimental conditions were set to 3 accumulations of 10 seconds, with a laser power of 2 mW. Measurements used a 20× objective and a spectrometer aperture of 50 μm.

**X-ray photoelectron spectroscopy (XPS).** XPS spectra were recorded on an S-Probe Monochromatized XPS spectrometer using an Al Kα source with a photon energy of 1486.6 eV. The spectra are shown in Figure S6. Further data treatment was carried out in CasaXPS. Binding energies were referenced to C 1s at 284.8 eV. Atomic ratios were calculated from background-subtracted integrated areas using the full-doublet RSFs for the metal 4f and Se 3d signals and the O 1s RSF listed in Tables S1a-b.

**Optical Absorption Spectroscopy.** The ground-state optical absorption spectra of the $HfSe_2$ and $ReSe_2$ dispersions were recorded using a double-beam PerkinElmer Lambda 1050 UV-vis spectrometer, equipped with an integrating sphere. In this way both the scattered and transmitted light are detected from which the fraction absorbed light can be determined directly.

**Transient Absorption (TA) Spectroscopy.** Transient absorption (TA) measurements on dispersions of liquid-phase exfoliated samples in NMP were carried out, using the experimental setup and procedure described previously,[10-12] and summarized below. A Yb: KGW oscillator (Light Conversion, Pharos SP) produces 180-fs pulses at 1028 nm with a 5 kHz repetition rate. Tunable pump wavelengths (330–1330 nm) were generated by directing the fundamental beam into an optical parametric amplifier equipped with a second-harmonic module (Light Conversion, Orpheus). A small fraction of the 1028 nm output was focused into a sapphire crystal to generate a broadband probe continuum spanning 480–1600 nm. The pump beam was modulated at 2.5 kHz using a mechanical chopper that transmitted every second pulse. Pump and probe beams were overlapped on the sample with a crossing angle of ~8°, and the pump–probe delay time, $\tau$, was controlled using a motorized delay stage. After interacting with the sample, the pump beam was discarded, and the probe beam was collected by a Helios detection system (Ultrafast Systems). The pump-induced change of the TA signal at probe photon energy, $E$, was determined according to $\Delta A(E,\tau) = \log_{10}\left(\frac{I_{off}(E)}{I_{on}(E,\tau)}\right)$, where $I_{on}$ and $I_{off}$ are the number of transmitted probe photons with and without photoexcitation, respectively. During all measurements, pump and probe polarizations were set orthogonal to minimize pump-induced scattering at the detector. Probe-chirp corrections were applied by polynomial fitting. The pump fluence was determined by measuring the beam profile with a camera-based beam profiler (Thorlabs, BC106-VIS).

## SUPPORTING FIGURES

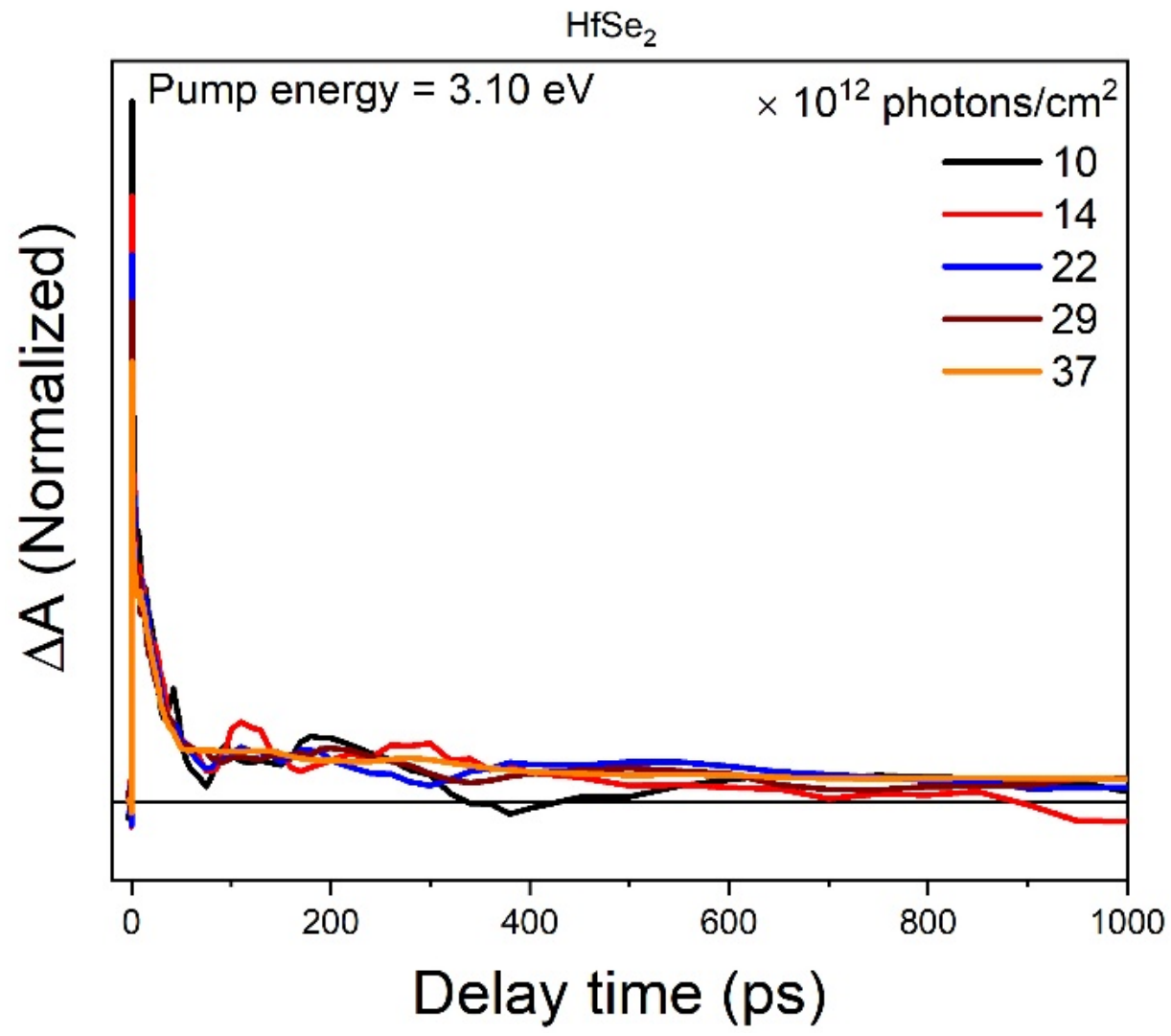


**Figure S1.** Decay kinetics of normalized TA signals for $HfSe_2$ probed at 1.86 eV for absorbed pump fluences as indicated, following photoexcitation at 3.10 eV. The similar kinetics at different pump fluences implies that charge carrier decay by first-order electron-hole recombination or trapping rather than higher-order (Auger) processes.

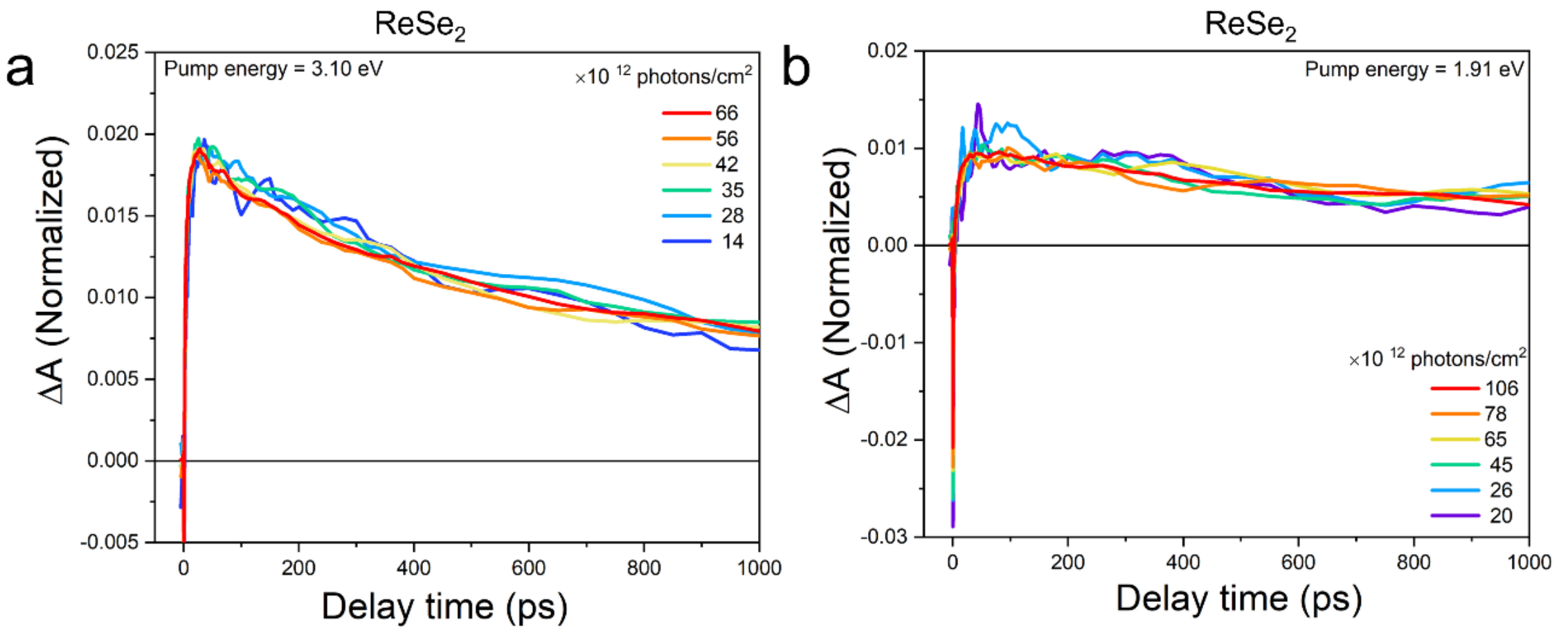


**Figure S2.** Normalized decay kinetics for $ReSe_2$ probed at the isosbestic point in the TA spectrum (photon energy 1.305 eV) after (a) pump pulses with photon energies of 3.10 eV and (b) 1.91 eV with different fluences, as indicated.

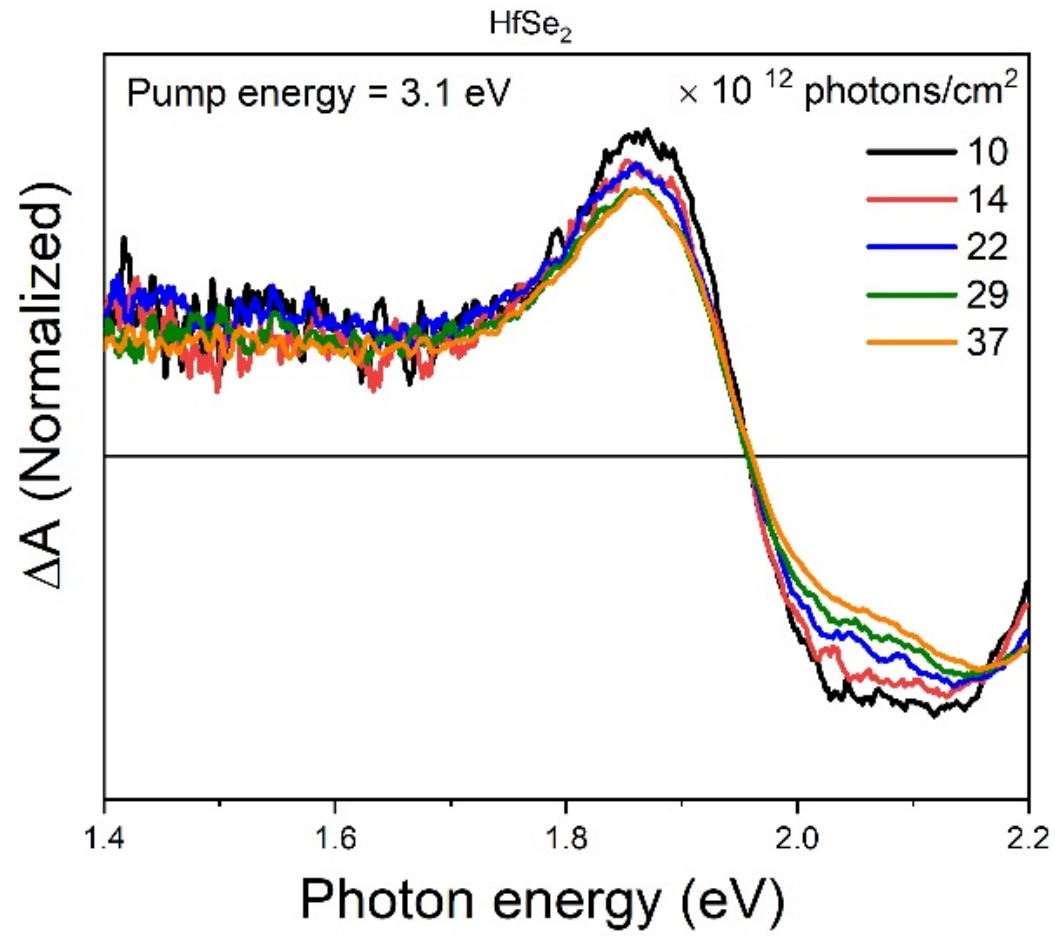


**Figure S3.** Normalized TA spectra of $HfSe_2$ for absorbed pump fluences as indicated and averaged over a delay time interval of 20-50 ps after photoexcitation at 3.10 eV.

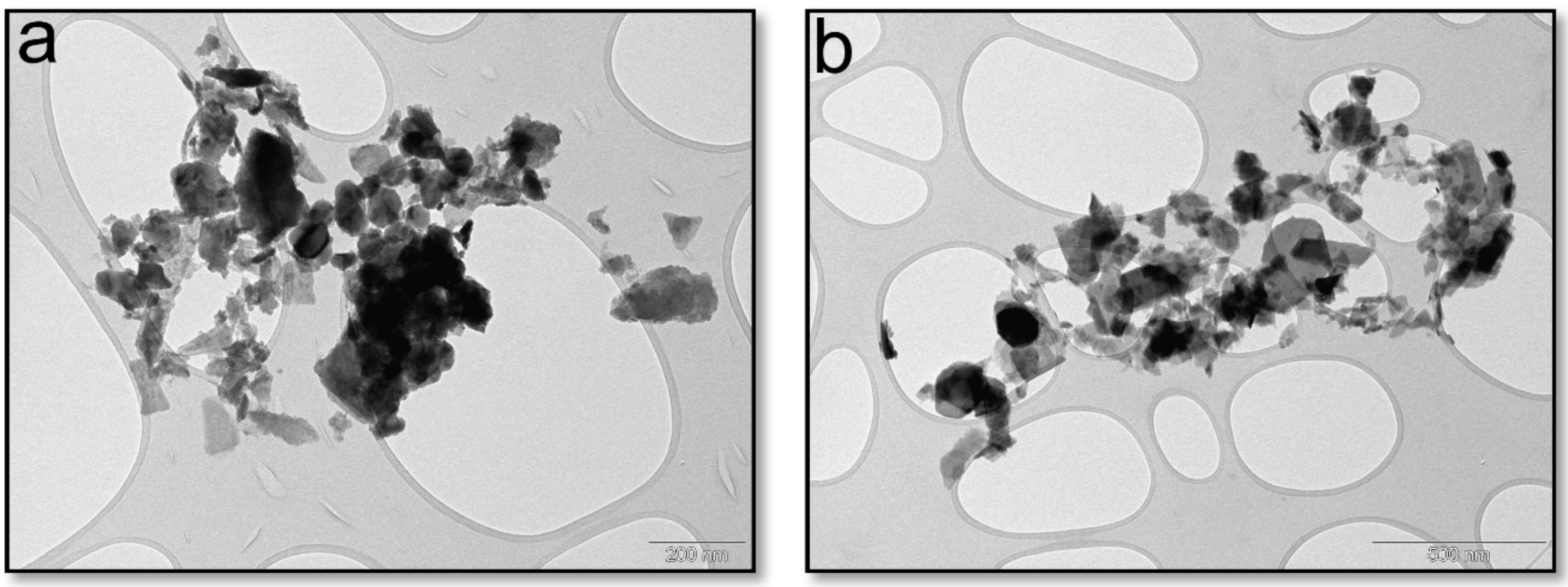


**Figure S4.** TEM images of exfoliated (a) $HfSe_2$ (scale bar is 200 nm) and (b) $ReSe_2$ (scale bar is 500 nm). The TEM images show flakes with varying contrast and overlapping regions, consistent with samples containing multilayer material.

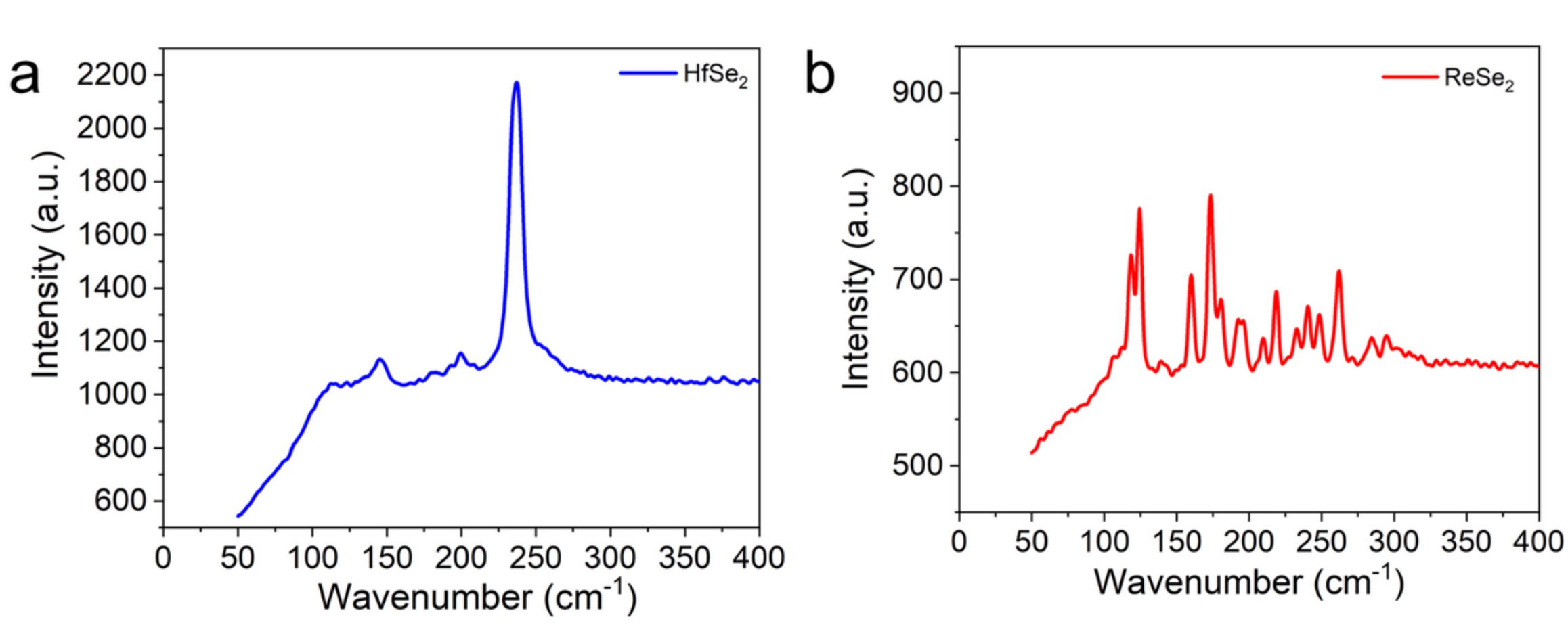


**Figure S5.** Raman spectra of exfoliated (a) $HfSe_2$ and(b) $ReSe_2$. Raw intensities are shown without baseline subtraction or smoothing. The $HfSe_2$ spectrum shows features near 145 and 200 $cm^{-1}$ due to the expected $E_g$ and $A_{1g}$ modes of 1T-$HfSe_2$, alongside a dominant selenium-related contribution.[13, 14] The $ReSe_2$ spectrum shows the characteristic multiplet of triclinic 1T'-$ReSe_2$.[15]

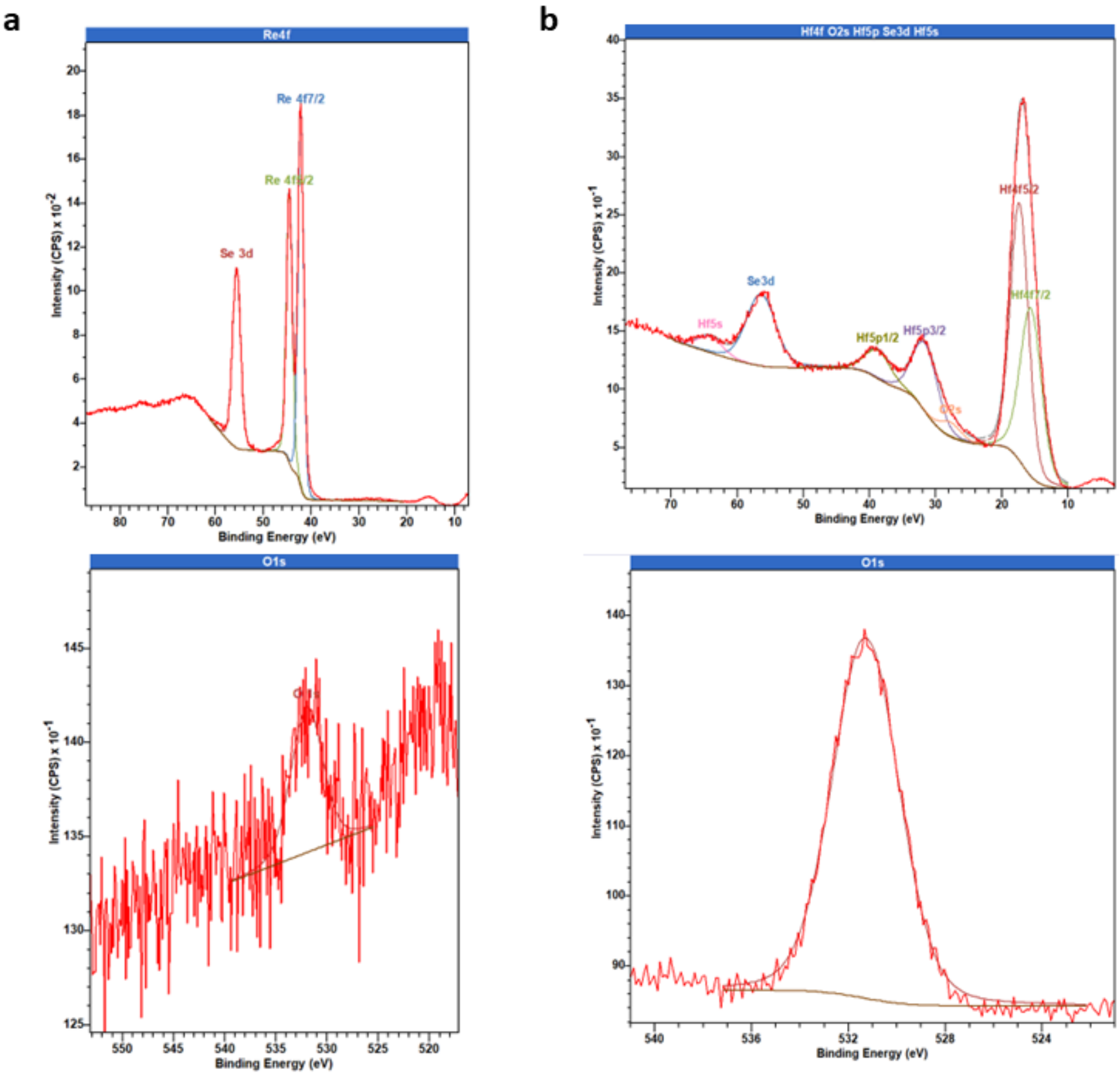


**Figure S6**. XPS spectra of liquid-phase exfoliated $ReSe_2$ and $HfSe_2$, showing the metal 4f, Se 3d and O 1s regions. Binding energies were referenced to C 1s at 284.8 eV. Integrated peak areas and relative sensitivity factors used to estimate the surface atomic ratios are listed in Table S1. Oxygen signals may include contributions from oxidation, adsorbates, residual solvent and the substrate.

**Discussion of XPS data.** $ReSe_2$ exhibits a Re 4f doublet at 42.2 and 44.6 eV and a Se 3d signal near 55.6 eV. The Re:Se atomic ratio of 1:1.95 is close to stoichiometric $ReSe_2$. An O 1s signal near 531 eV gives O/Re ≈ 0.26, although the weak oxygen signal makes this estimate sensitive to noise and background selection. No additional oxidised-Re component is clearly resolved, but minor surface oxidation cannot be excluded.

For $HfSe_2$, the Hf 4f doublet at 15.7 and 17.4 eV, Se 3d signal near 56.3 eV, and O 1s signal near 531.5 eV give an estimated Hf:Se:O ratio of approximately 1:0.90:3.70. This indicates a selenium-poor, oxygen-rich measured surface and suggests surface alteration. Total oxygen cannot be assigned exclusively to Hf oxide, and broadening and charging uncertainty prevent reliable quantification of the oxide fraction. XPS and Raman samples were exposed to

air during preparation, whereas TA dispersions were handled and measured under oxygen-free nitrogen conditions.

**Table S1a.** XPS binding energies, relative sensitivity factors (RSFs), integrated peak areas and RSF-corrected areas for $ReSe_2$. Normalisation to Re gives an estimated surface atomic ratio of Re:Se:O ≈ 1:1.95:0.26. Metal 4f and Se 3d areas and RSFs refer to the complete doublets.

| Peak | BE (eV) | RSF | area | area/RSF |
|---|---|---|---|---|
| **Re 4f (doublet)** | 42.2 / 44.6 | 11.08 | 4672 | 422 |
| **Se 3d** | 55.6 | 2.294 | 1883 | 821 |
| **O 1s** | 531 | 2.93 | 327 | 111 |

**Table S1b.** XPS binding energies, relative sensitivity factors (RSFs), integrated peak areas and RSF-corrected areas for $HfSe_2$. Normalisation to Hf gives an estimated surface atomic ratio of Hf:Se:O ≈ 1:0.90:3.70. Metal 4f and Se 3d areas and RSFs refer to the complete doublets. Total oxygen includes possible contributions beyond Hf oxide and does not directly quantify the oxide fraction.

| Peak | BE (eV) | RSF | area | area/RSF |
|---|---|---|---|---|
| **Hf 4f (doublet)** | 15.7 / 17.4 | 7.52 | 1335 | 178 |
| **Se 3d** | 56.3 | 2.294 | 368 | 160 |
| **O 1s** | 531.5 | 2.93 | 1924 | 656 |

## SUPPORTING REFERENCES


(1) Gonze, X.; Amadon, B.; Antonius, G.; Arnardi, F.; Baguet, L.; Beuken, J.-M.; Bieder, J.; Bottin, F.; Bouchet, J.; Bousquet, E.; et al. The Abinitproject: Impact, environment and recent developments. *Comput. Phys. Commun.* **2020**, *248*, 107042.

(2) McTaggart, F. K.; Wadsley, A. D. The sulphides, Selenides, and Tellurides of Titanium, Zirconium, Hafnium, and Thorium. I. Preparation and Characterization. *Aust. J. Chem.* **1958**, *11*, 445–457.

(3) Yue, R.; Barton, A. T.; Zhu, H.; Azcatl, A.; Pena, L. F.; Wang, J.; Peng, X.; Lu, N.; Cheng, L.; Addou, R.; et al. $HfSe_2$ Thin Films: 2D Transition Metal Dichalcogenides Grown by Molecular Beam Epitaxy. *ACS Nano* **2015**, *9* (1), 474–480.

(4) Eknapakul, T.; Fongkaew, I.; Siriroj, S.; Vidyasagar, R.; Denlinger, J. D.; Bawden, L.; Mo, S. K.; King, P. D. C.; Takagi, H.; Limpijumnong, S.; et al. Nearly-Free-Electron System of Monolayer Na on The Surface of Single-Crystal $HfSe_2$. *Phys. Rev. B* **2016**, *94* (20), 201121.

(5) Alcock, N. W., Kjekshus, A. The Crystal Structure of $ReSe_2$. *Acta Chem. Scand.* **1965**, *19*, 79–94.

(6) Jiang, H. Structural and Electronic Properties of $ZrX_2$ and $HfX_2$ (X = S and Se) from First Principles Calculations. *J. Chem. Phys.* **2011**, *134* (20), 204705.

(7) Ho, C. H.; Huang, C. E. Optical Property of the Near Band-Edge Transitions in Rhenium Disulfide and Diselenide. *J. Alloys Compd.* **2004**, *383* (1), 74–79.

(8) Weerdenburg, S.; Singh, N.; van der Laan, M.; Kinge, S.; Schall, P.; Siebbeles, L. D. A. New Theoretical Model to Describe Carrier Multiplication in Semiconductors: Explanation of Disparate Efficiency in $MoTe_2$ versus PbS and PbSe. *J. Phys. Chem. C* **2024**, *128* (9), 3693–3702.

(9) Schiettecatte, P.; Rousaki, A.; Vandenabeele, P.; Geiregat, P.; Hens, Z. Liquid-Phase Exfoliation of Rhenium Disulfide by Solubility Parameter Matching. *Langmuir* **2020**, *36* (51), 15493–15500.

(10) Ghosh, G.; Carey, T.; Furxhiu, S.; Weerdenburg, S.; Singh, N.; van der Laan, M.; Branchett, S. E.; Jaspers, S.; Suijkerbuijk, J. W.; Lipilin, F.; et al. Carrier Multiplication and Photoexcited Many-Body States in Solution-Processed 2H-$MoSe_2$. *ACS Nano* **2025**, *19* (10), 10347–10358.

(11) Schiettecatte, P.; Poonia, D.; Tanghe, I.; Maiti, S.; Failla, M.; Kinge, S.; Hens, Z.; Siebbeles, L. D. A.; Geiregat, P. Unraveling the Photophysics of Liquid-Phase Exfoliated Two-Dimensional $ReS_2$ Nanoflakes. *J. Phys. Chem. C* **2021**, *125* (38), 20993–21002.

(12) Grimaldi, G.; Geuchies, J. J.; van der Stam, W.; du Fossé, I.; Brynjarsson, B.; Kirkwood, N.; Kinge, S.; Siebbeles, L. D. A.; Houtepen, A. J. Spectroscopic Evidence for the Contribution of Holes to the Bleach of Cd-Chalcogenide Quantum Dots. *Nano Lett.* **2019**, *19* (5), 3002–3010.

(13) Ly, C. Y.; Vong, C.; Sriv, T.; Cheong, H. Raman Spectroscopy of Atomically Thin $HfX_2$ (X=S, Se). *2D Materials* **2024**, *11* (4), 045013.

(14) Sahota, S.; Chircă, I.; Burton, O. J.; Yu, H.; Rimmer, M.; Yang, J.; Park, K.; Summers, A.; Mertdinc-Ulkuseven, S.; Lindley, M.; et al. Se Nanowire Crystal Formation via Oxidation of 2D $HfSe_2$: A Solid-State, In Situ Reaction Coupling for Heterogeneous Integration Technologies. *ACS Appl. Nano Mater.* **2025**, *8* (15), 7608–7615.

(15) Wolverson, D.; Crampin, S.; Kazemi, A. S.; Ilie, A.; Bending, S. J. Raman Spectra of Monolayer, Few-Layer, and Bulk $ReSe_2$: An Anisotropic Layered Semiconductor. *ACS Nano* **2014**, *8* (11), 11154–11164.
